# The Choice of the Filtering Method in Microarrays Affects the Inference Regarding Dosage Compensation of the Active X-Chromosome

**DOI:** 10.1371/journal.pone.0023956

**Published:** 2011-09-01

**Authors:** Raphaële Castagné, Maxime Rotival, Tanja Zeller, Philipp S. Wild, Vinh Truong, David-Alexandre Trégouët, Thomas Munzel, Andreas Ziegler, François Cambien, Stefan Blankenberg, Laurence Tiret

**Affiliations:** 1 INSERM UMRS 937, Pierre and Marie Curie University (UPMC, Paris 6) and Medical School, Paris, France; 2 II. Medizinische Klinik und Poliklinik, Johannes-Gutenberg Universität Mainz, Universitätsmedizin, Mainz, Germany; 3 Institut für Medizinische Biometrie und Statistik, Universität zu Lübeck, Universitätsklinikum Schleswig-Holstein, Lübeck, Germany; Florida State University, United States of America

## Abstract

**Background:**

The hypothesis of dosage compensation of genes of the X chromosome, supported by previous microarray studies, was recently challenged by RNA-sequencing data. It was suggested that microarray studies were biased toward an over-estimation of X-linked expression levels as a consequence of the filtering of genes below the detection threshold of microarrays.

**Methodology/Principal Findings:**

To investigate this hypothesis, we used microarray expression data from circulating monocytes in 1,467 individuals. In total, 25,349 and 1,156 probes were unambiguously assigned to autosomes and the X chromosome, respectively. Globally, there was a clear shift of X-linked expressions toward lower levels than autosomes. We compared the ratio of expression levels of X-linked to autosomal transcripts (X∶AA) using two different filtering methods: 1. gene expressions were filtered out using a detection threshold irrespective of gene chromosomal location (the standard method in microarrays); 2. equal proportions of genes were filtered out separately on the X and on autosomes. For a wide range of filtering proportions, the X∶AA ratio estimated with the first method was not significantly different from 1, the value expected if dosage compensation was achieved, whereas it was significantly lower than 1 with the second method, leading to the rejection of the hypothesis of dosage compensation. We further showed in simulated data that the choice of the most appropriate method was dependent on biological assumptions regarding the proportion of actively expressed genes on the X chromosome comparative to the autosomes and the extent of dosage compensation.

**Conclusion/Significance:**

This study shows that the method used for filtering out lowly expressed genes in microarrays may have a major impact according to the hypothesis investigated. The hypothesis of dosage compensation of X-linked genes cannot be firmly accepted or rejected using microarray-based data.

## Introduction

It is widely admitted that in mammals, X-linked genes are upregulated to ensure balanced expression between the X chromosome, present in a single active copy per cell, and autosomes, present in two copies [1]. The hypothesis of dosage compensation first proposed by Ohno in 1967 [2] was supported by recent microarray studies showing that X-linked genes were expressed at similar levels to autosomal genes in mice and humans [3]–[5]. However, the molecular mechanism responsible for this compensation is still not understood [6]. The absence of mechanistic interpretation, coupled to the lack of dosage compensation in other taxa [7], [8], have spurred speculation about this phenomenon.

Recently, Ohno's hypothesis was challenged by a study using RNA sequencing (RNA-Seq) data showing that the ratio of the median expression level of X-linked genes to that of autosomal genes (X∶AA) was significantly lower than 1 in different human and mouse tissues [9]. The authors attributed the difference between their findings and previous ones to the fact that RNA-Seq is much more sensitive than microarray to detect small expression differences [9]–[11] and that microarray studies are likely to be biased towards an over-estimation of X-linked expression levels as a consequence of the filtering of genes considered to be under the detection threshold of microarray [9]. This controversial finding led us to question the method conventionally used for the analysis of microarray-based expression data. Using data from a large-scale expression study in human monocytes [12], we showed that according to the method used for filtering out the genes prior to analysis, the inference regarding dosage compensation was in the opposite direction. A simulation study further demonstrated that the choice of the most appropriate filtering method was dependent on biological assumptions regarding the proportion of actively expressed genes on the X chromosome and on autosomes and the extent of dosage compensation. Although the limited sensitivity of microarrays does not allow one to go further in resolving this issue, the potential methodological bias arising from using a signal-threshold cutoff in microarray experiments should be kept in mind when comparing expression across loci.

## Results

### Filtering transcripts considered as undetected by microarrays may discard genes that show biologically relevant associations supporting cellular expression

In microarray studies, genes whose expression is not significantly different from the background signal are conventionally filtered out prior to analysis. These genes are often inappropriately considered as unexpressed in the cell type under study although they are only undetected. Recent studies based on RNA-Seq have shown that a fraction of the genes undetected by microarrays were actually expressed at low levels in the cells investigated [9], [10], [13]. This filtering based on a statistical detection criterion was justified in former small microarray studies which were mainly designed for discovering large expression differences between contrasted experimental conditions and therefore focused on highly expressed genes. However, it may be less appropriate in current large-scale transcriptomic studies which are more interested in characterizing the natural sources of variability of gene expression, such as genetic variations, environmental exposures, metabolic conditions, ageing or gender [12]. Actually, a gene expression level that is below the detection threshold of microarrays may be found to be related to a SNP or another relevant factor that provides biological evidence that the gene is expressed. This is a problem known as signal-to-noise in biology [14].

**Figure 1:**
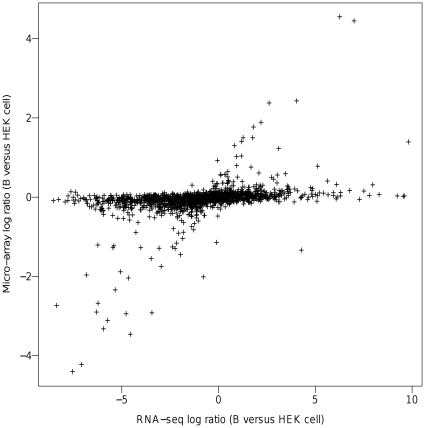
Comparison of differentially expressed genes (B versus HEK cells) by RNA sequencing and microarrays. Data are drawn from Sultan et al. [10] and genes detected by RNA-Seq (at least five reads) but not detected by microarrays (detection score <0.95) were selected (1,640 genes in total). The plot shows log2 ratios of expression in RNA-Seq (*x* axis) and microarrays (*y* axis).

To illustrate this issue, we re-analyzed the data of a previous study in which gene expression was simultaneously measured by microarray and RNA-Seq in two different cell lines, HEK and B cells [10]. In this study, RNA-Seq could detect 25% more gene expressions than could microarrays. When the authors focused on genes detected by both platforms and in both cell lines (n = 7,043), they showed that the differences of gene expression between HEK and B cells (measured by the log ratio of expression) strongly correlated across the two platforms (r = 0.88) in spite of a compression effect resulting in smaller ratios in microarrays. This result indicated that true biological differences between cell types were reproducibly found across platforms. Using the same dataset, we performed a similar analysis on the genes that were detected by RNA-Seq (at least five reads) but were undetected by microarray (detection score <0.95) (1,640 genes). As shown in Figure 1, there was a subset of genes lying along the diagonal in which differential expression between HEK and B cells strongly correlated across the two platforms. For these genes, which are likely to be truly differentially expressed between cell types, the difference could be detected by microarrays even though their expression level was considered not different from the background noise. This demonstrates that for genes below the detection level of microarrays, biologically relevant signals can be found that indicate that the gene is expressed.

### Testing the hypothesis of dosage compensation of X-linked genes in human monocytes

To investigate the hypothesis of dosage compensation of X-linked genes, we used expression data from the Gutenberg Heart Study (GHS), a population-based study in which the transcriptome of circulating monocytes was assessed in 1,467 unrelated subjects (51.1% of men) by microarray using the *Illumina* HT-12 v3 BeadChip [12]. After removing probes with a bad quality score according to ReMOAT [15], 25,349 probes were unambiguously assigned to the autosomes and 1,156 probes to the X chromosome. Analyses were performed at the probe level and for simplicity the term of “transcript” was used to denote a unique probe-hybridization product (although in few cases the same transcript could be targeted by several probes or conversely, a same probe could target several transcripts). Analyses were performed in males and females separately.

**Figure 2:**
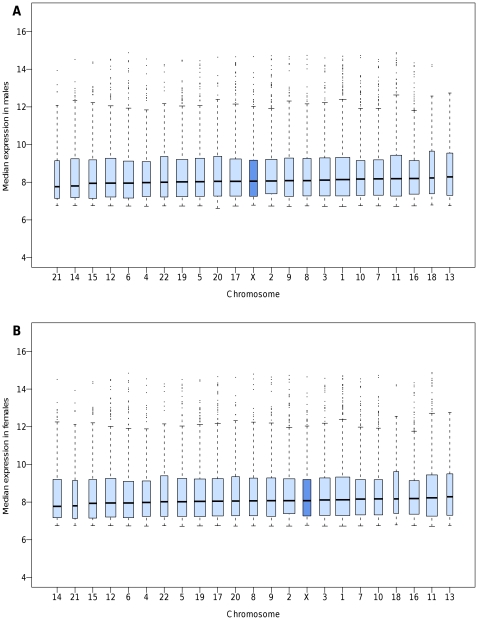
Box plots of the median expression levels in human monocytes according to chromosome when selecting the transcripts detected in at least 95% of individuals. (**A**) Males and (**B**) Females.

As usually performed in microarray experiments, we first selected the transcripts whose expression was detected in ≥95% of samples. This filtering resulted in the selection of 10,896 autosomal and 360 X-linked transcripts, the vast majority of them being detected in both genders. The proportion of transcripts filtered out prior to analysis was 57.5% as a whole, but it was much higher on the X chromosome than on autosomes (68.9% vs 57.0%, *P*<10^−6^). In the subset of selected transcripts, the median level of expression of X-linked transcripts was not significantly different from that of autosomal transcripts in both sexes (7.68 vs 7.87, *P* = 0.09 in males; 7.71 vs 7.85, *P* = 0.12 in females). As previously reported [5], expression levels of X-linked genes fell within the global range of autosomes, suggesting that dosage compensation of X-linked genes was globally achieved in both sexes (Figures 2A and 2B).

**Figure 3:**
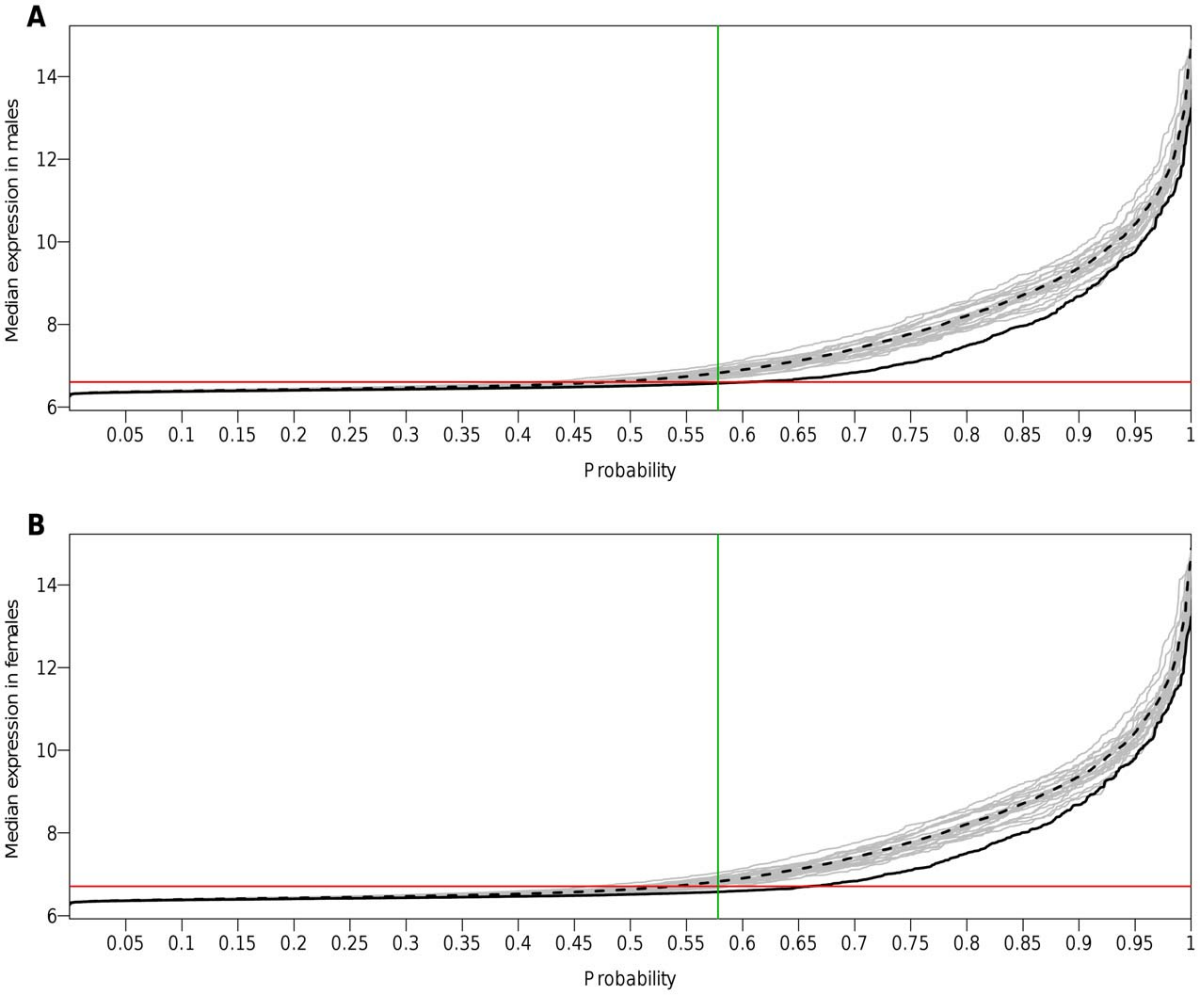
Quantile functions of median expression levels of X-linked and autosomal transcripts in human monocytes. (**A**) **Males and** (**B**) **Females:** X-linked transcripts are shown by the plain black curve, autosomal transcripts by the dashed black curve and each autosome by an individual grey curve. For a probability *p* (*x*-axis), the *y*-axis shows the median expression level below which *p*×100% of transcripts fall. The horizontal red line corresponds to the filtering performed when selecting transcripts detected in ≥95% of individuals. The vertical green line corresponds to excluding equal proportions (57.5%) of the less expressed genes on the X chromosome and on autosomes separately.

However, as shown by the quantile functions of expression levels plotted separately for the X chromosome and for autosomes, there was a clear shift of X-linked expressions towards lower levels than autosomes in both sexes (Figures 3A and 3B). As a consequence, taking a uniform detection threshold for the X and for autosomal chromosomes led to a greater truncation of transcripts on the X chromosome than on autosomes (as shown by the horizontal plain red line in Figures 3A and 3B) resulting in an over-estimation of X-linked expressions in the subset of genes kept for analysis. In order to circumvent this potential bias, we compared expression levels between X-linked and autosomal transcripts after excluding equal proportions of the less expressed transcripts on the X and on autosomes separately (as shown by the vertical green line in Figures 3A and 3B). When excluding the same proportion of transcripts as above (57.5%), but considering this time the X chromosome and autosomes separately, the difference of median expression levels between X-linked and autosomal transcripts became highly significant in both sexes (6.82 vs 7.91, *P*<10^−31^ in males; 6.85 vs 7.91, *P*<10^−30^ in females).

**Figure 4:**
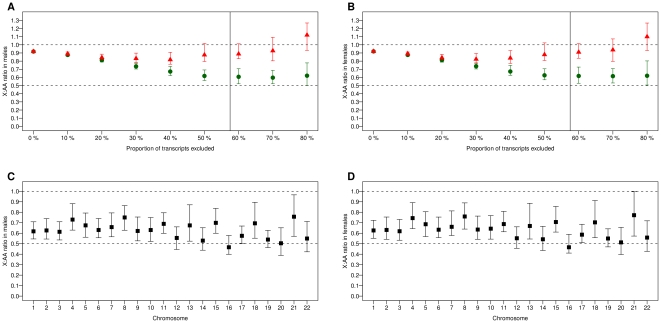
Comparison of expression levels between the X chromosome and autosomes in human monocytes. (**A**) **Males and** (**B**) **Females:** The graph plots the X∶AA ratio of median expression of X-linked genes to autosomal genes according to the proportion of transcripts filtered out prior to analysis, using either a uniform threshold (red triangles) or individual thresholds on the X and on autosomes (green circles). Error bars show the 95% bootstrap confidence intervals. The horizontal dashed lines show the ratios expected if there was no dosage compensation (X∶AA = 0.5) or full compensation (X∶AA = 1). The vertical line corresponds to the proportion of genes filtered out when using a detection score ≥95%. (**C**) **Males and** (**D**) **Females:** X∶AA ratios when the X is compared to individual autosomes and the same proportion of transcripts (50%) is filtered on the X and on each autosome.

We then compared the X∶AA ratio of expression level of X-linked transcripts to autosomal transcripts when filtering either a global proportion of the lowest gene expressions irrespective of their chromosome location (model 1 which is equivalent to considering a uniform detection threshold) or an equal proportion of the lowest gene expressions on the X and on autosomes separately (model 2). In order to investigate the impact of the filtering threshold on the estimation of the X∶AA ratio, we varied the proportion of filtered transcripts, which corresponded to moving the horizontal red lines from the bottom to the top (model 1) or the vertical green lines from the left to the right (model 2) in Figures 3A and 3B. As shown in Figures 4A and 4B, the X∶AA ratio was always higher when using a uniform filtering threshold not depending on the gene chromosomal location (model 1, red triangles) than when using a chromosome-specific threshold (model 2, green circles). The difference between the two X∶AA estimates increased as the filtering became more stringent as a result of a greater truncation in model 1 of lowly expressed genes on the X chromosome than on autosomes. When the proportion of genes filtered out prior to analysis was greater or equal to 50%, the X∶AA ratio estimated when taking a uniform filtering threshold was no longer significantly different from 1, the value expected if there was dosage compensation, whereas it was significantly lower than 1 when taking a chromosome-specific threshold. Results were very similar in males and females (Figure 4A and 4B).

We also estimated the X∶AA ratio for each autosome individually after filtering out the same proportion (50%) of the lowest gene expressions on each chromosome. For all autosomes, the X∶AA ratio was significantly lower than 1 (Figure 4C and 4D). Worthy of note, the X∶AA ratio associated to chromosome 21, which was the highest in human liver RNA-seq data [9], was also the highest in human monocytes.

The number of probes per gene was slightly higher on the X chromosome than on autosomes (1.64 vs 1.51). To check whether this difference might have an impact on the results, we repeated the analysis using the most variable probe for each gene or using average expression across probes. Although the X∶AA ratio tended to be slightly higher than when focusing on probes, the same trends were observed (Figure S1).

### Dosage compensation of X-linked genes in other human and mouse tissues

**Figure 5:**
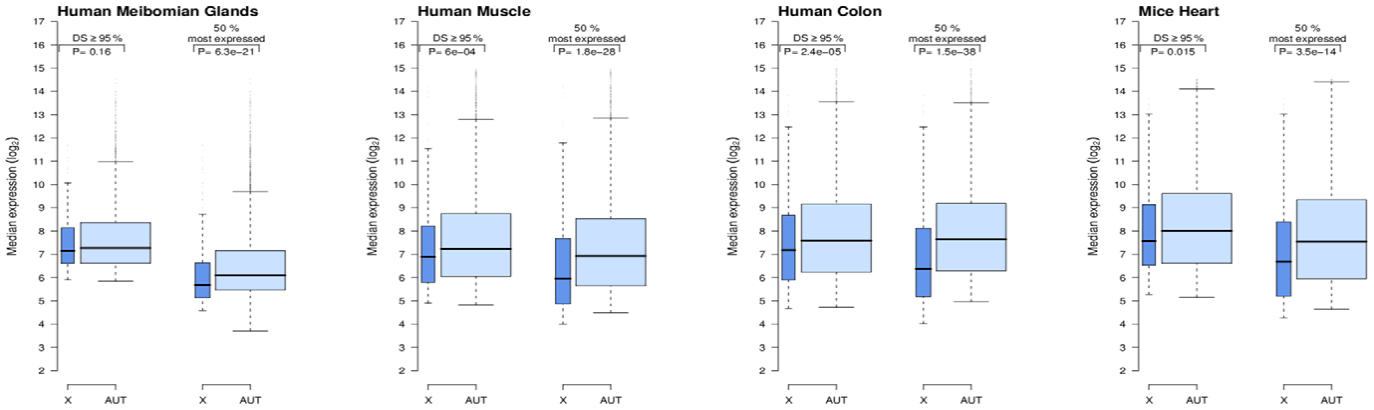
Expression levels of X-linked and autosomal transcripts in different human and mouse tissues. The graph shows boxplots of expression levels either when filtering the genes according to a uniform detection threshold (detection score (DS) ≥95%) or when excluding the 50% lowest gene expressions separately on the X chromosome and on autosomes (AUT).

Because the expression of genes is known to be tissue specific, we checked whether the same observation could be made from other tissues. For this purpose, we analyzed publicly available microarray expression data from three different human tissues, the meibomian glands (access number GSE17822), the muscle (access number GSE20319) and the colon (access number GSE26305). For each tissue dataset, we selected either the transcripts for which the detection score was greater than 95% (filtering not depending of chromosome), or the same proportion (arbitrarily taken to 50%) of the most highly expressed transcripts separately on the X chromosome and on autosomes. Whatever the tissue under consideration, the differences of expression levels between X-linked and autosomal genes were always more pronounced when filtering out separately genes on the X and on autosomes (Figure 5). The same observation was made for microarray expression data from mouse heart tissue (access number GSE27689) (Figure 5). Unlike what had been previously reported from RNA-Seq data [9], we did not observe a lower X∶AA ratio in mice than in humans in this mouse dataset (Figure S2).

### Comparison with genes submitted to genomic imprinting

In mammals, genomic imprinting affects a small proportion (<1%) of autosomal genes and results in the expression of only one allele inherited from the father or the mother. In terms of expressed alleles, imprinted genes are thus comparable to X-linked genes which are submitted to inactivation of one of the two alleles. We hypothesized that imprinted genes may exhibit a similar pattern of expression to the one observed in X-linked genes.

**Figure 6:**
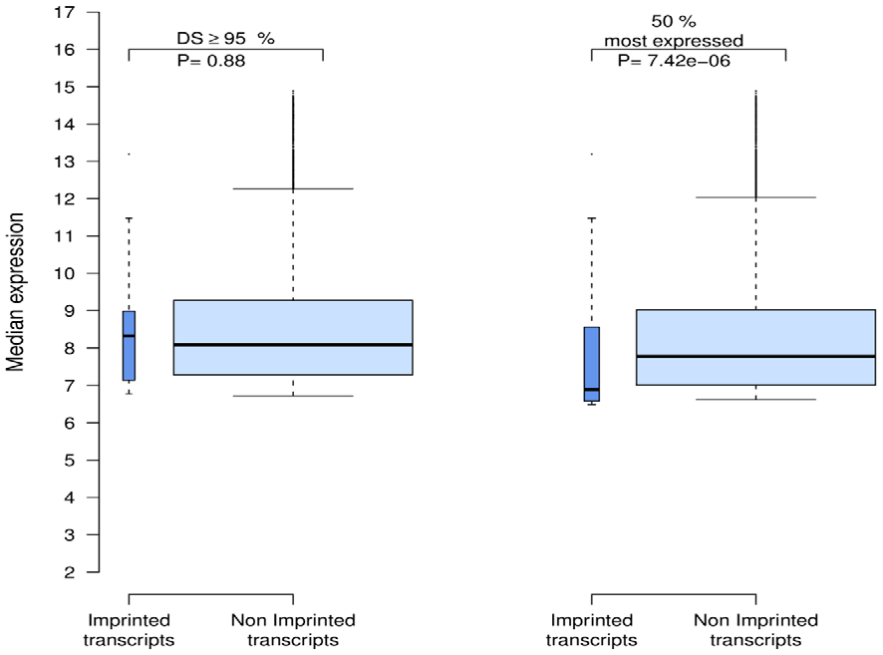
Expression levels of imprinted and non-imprinted autosomal transcripts in human moncoytes. The graph shows boxplots of expression levels either when filtering the genes according to a uniform detection threshold (detection score (DS) ≥95%) or when excluding the 50% lowest gene expressions separately in imprinted and non-imprinted genes.

To test this hypothesis, we compared the levels of imprinted transcripts to those of non-imprinted transcripts in the GHS dataset. Among the 10,806 well-annotated autosomal probes, 97 probes (listed in Table S1) corresponded to genes that were reported to be submitted to imprinting in two databases (http://igc.otago.ac.nz/home.html and http://www.geneimprint.com/). When first selecting the transcripts whose expression was detected in ≥95% of samples, the proportion of transcripts filtered out prior to analysis was much higher for imprinted than for non-imprinted transcripts (72.2% vs 57.3%, *P*<10^−6^). In the subset of selected transcripts, the median level of expression of imprinted transcripts was not significantly different from that of non-imprinted transcripts (8.32 vs 8.08, *P* = 0.88). By contrast, when selecting a similar proportion (arbitrarily taken to 50%) of the most highly expressed transcripts separately among imprinted and non-imprinted genes, expression levels were significantly lower in imprinted transcripts than in non-imprinted ones (6.89 vs 7.78, *P*<0.001) (Figure 6).

### Simulation study

By filtering out the same proportion of genes on each chromosome, we implicitly make the assumption that the same proportion of genes are actively expressed on the X chromosome and on autosomes, an assumption that may not be true, at least in all tissues. We addressed this issue using the gene expression dataset from HEK and B cells already used above [7]. In HEK cells, the proportions of genes actively expressed (i.e. detected by RNA-Seq) on autosomes and on the X chromosome were relatively similar (81.2% vs 76.8%, *P* = 0.02), whereas in B cells, these proportions were globally lower and differed more drastically between autosomes and the X (70.0% vs 58.8%, *P* = 2.4×10^−6^). These results suggested that the assumption of equal proportionality of expressed genes by chromosome may not hold in all cell types.

**Figure 7:**
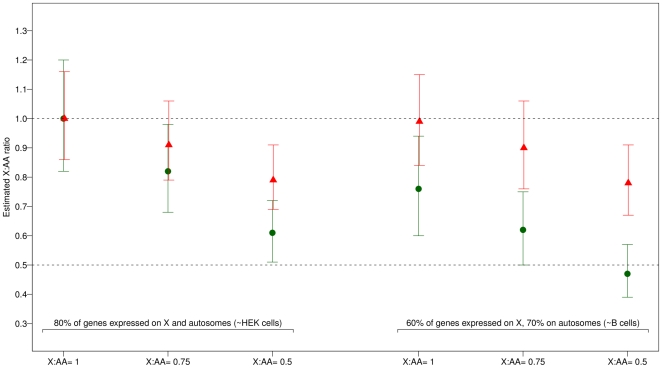
X∶AA ratio estimated from data simulated under different models of dosage compensation and assuming variable proportions of expressed genes on the X and on autosomal chromosomes (see legend of *x* axis). In all cases, 50% of transcripts were filtered out prior to analysis, using either a uniform threshold (red triangles) or individual thresholds on the X and on autosomes (green circles).

We further explored the impact of different assumptions using simulations. We simulated expression data assuming either an equal proportion of actively expressed genes on autosomes and on the X (80% as in HEK cells), or a lower proportion on the X than on autosomes (60% vs 70% as in B cells). For each cell type, we additionally assumed that there was either full dosage compensation (X∶AA ratio = 1), partial compensation (X∶AA ratio = 0.75) or complete lack of compensation (X∶AA ratio = 0.5). Expression levels were simulated according to the model proposed by Lin et al. [16] with parameters based on the empirical values observed in the GHS dataset (see Methods). The X∶AA ratio was then estimated using the two different methods for filtering out gene expressions (referred to as “uniform filtering” and “chromosome-specific filtering”, respectively). For both methods, the proportion of filtered genes was fixed at 50% and the null hypothesis tested was that of full dosage compensation. Results of the simulation study are presented in Figure 7.

Under the assumption of equal proportions of expressed genes (as in HEK cells), both methods correctly estimated the X∶AA ratio to 1 when there was full compensation. When there was partial compensation, the uniform filtering method did not allow the rejection of the null hypothesis whereas the chromosome-specific filtering method did. In case of complete lack of compensation, both methods allowed the rejection of the null hypothesis.

Under the assumption of different proportions of expressed genes (as in B cells), the chromosome-specific filtering method tended to globally under-estimate the true X∶AA ratio, leading to falsely reject the null hypothesis of compensation when this latter was true, whereas in that case the uniform filtering method correctly estimated the X∶AA ratio. For partial compensation, again the uniform filtering method did not allow the rejection of the null hypothesis whereas the chromosome-specific filtering method did. Both methods allowed the rejection of the null hypothesis when there was no compensation.

## Discussion

The present study addresses an important issue concerning the potential biases arising from the method of filtering genes in expression studies. In microarray studies, it is generally advocated to select only the genes whose expression is detected in the majority of samples, the remaining genes being considered as not expressed in the cell type under study. However, with the advent of more sensitive techniques like RNA-Seq, as well as the greater power of contemporary transcriptomic studies, it is realized that many genes considered as unexpressed in microarray experiments are actually expressed at low levels, or only in a fraction of the population, for example when expression is modulated by a genetic or an environmental factor.

This issue is particularly critical when comparing expression across loci, as for the analysis of dosage compensation comparing genes on different chromosomes. We showed that analyzing data by conventional filtering methods of microarrays led to the conclusion that in human monocytes, expression levels of X-linked genes did not differ from those of autosomal genes. This result would support the hypothesis of dosage compensation of X-linked genes, as reported by previous microarray studies [3]–[5]. On the other hand, if there is no dosage compensation, as recently suggested by RNA-Seq data [9], expression levels of X-linked genes are expected to be lower than those of autosomal genes. Applying the standard filtering threshold of microarrays would then bias the results by excluding a disproportionate number of lowly expressed genes on the X chromosome. When applying a chromosome-specific filtering threshold to circumvent this bias, the hypothesis of dosage compensation was no longer supported by the data. However, the use of a chromosome-specific threshold implicitly rests on the assumption that the same proportion of genes are expressed on the X chromosome and on autosomes, an assumption which may hold in some cell types but not in others, as suggested by the comparison of HEK and B cells. Depending on the underlying biological reality, we showed by simulations that either method of filtering might lead to false inference. This potential bias should be kept in mind in analysis of microarray experiments. Hopefully, this should be no longer an issue with the development of highly sensitive technologies for the quantification of transcript abundance such as RNA-Seq. Interestingly, in a recent RNA-Seq study on mouse Th2 cells, two distinct groups of genes could be detected, one group of lowly expressed and putatively non-functional mRNAs, and the other group of highly expressed and functional mRNAs [13]. This suggests that the distinction between expressed and non-expressed genes may be even more subtle than initially thought.

## Materials and Methods

### Ethic statement

The study protocol and drawing of the blood sample have been approved by the local ethics committee and by the local and federal data safety commissioners (Ethik-Kommission der Landesärztekammer Rheinland-Pfalz 22/03/2007 Number 837.020.07 (5555)). All subjects included signed an informed consent.

### Study Population

The study has been described in details elsewhere [12]. Study participants of both sexes aged 35–74 yr, were successively enrolled into the Gutenberg Heart Study (GHS), a community-based single centre cohort study conducted in the Rhein-Main region in western mid-Germany. All subjects were of European descent. Individuals for whom we found a discrepancy between the phenotypic gender and the sex inferred from expression of Y-linked transcripts were excluded, leaving 1,467 individuals for analysis (750 men and 717 women).

### Genome-wide expression

Genome-wide expression profiles were assessed from peripheral blood monocytes. Separation of monocytes was conducted within 60 min after blood collection by negative selection using RosetteSep Monocyte Enrichment Cocktail (StemCell Technologies, Vancouver, Canada). Total RNA was extracted the same day using Trizol extraction and purification by silica-based columns. Expression profiles were assessed using the Illumina HT-12 v3 BeadChip. The pre-processing of data was performed using Beadstudio. Values from probes with ≤1 bead were re-imputed using the SVD impute from the pcaMethods R package. Data were normalized using quantile normalization and VST transformation [16] as implemented in the lumi R package [17]. After removing probes with a bad quality score according to ReMOAT (http://remoat.sysbiol.cam.ac.uk), 25,349 and 1,156 probes were unambiguously assigned to the autosomes and the X chromosome, respectively.

### Statistical Analysis

Analyses were performed at the probe level unlike other specified. To select the transcripts that were detected in ≥95% of individuals, we used the detection *P*-values provided by the *Illumina* software and considered that a transcript was detected in a sample when the detection *P*-value for that sample was <0.05. Proportion of detected genes between the X chromosome and autosomes were compared using a Chi^2^ test with 1df. Median expression levels were compared between the X chromosome and autosomes using a Mann-Whitney U test. The X∶AA ratio was the ratio between median expression levels of X-linked genes to median expression levels of autosomal genes. The 95% bootstrap confidence interval was estimated by resampling 1000 times the datasets of X-linked and autosomal transcripts.

We estimated the X∶AA ratio using either a common filtering proportion of genes irrespective of the chromosomal location, or a proportion specific of the X/autosomal location. In the former case, we excluded the *k*% lowest transcripts among all transcripts, whereas in the latter case, we excluded the *k*% lowest X-linked transcripts and the *k*% lowest autosomal transcripts (*k* varying from 0% to 80%). For the comparison of the X chromosome to individual autosome, the same proportion of genes was excluded on each autosome and on the X.

### Simulations

We simulated expression levels in 25,349 autosomal transcripts and 1,156 X-linked transcripts under different hypotheses. For each transcript, expression level was simulated using the model proposed by Lin et al. [16]:
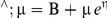
where 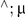 is the observed transcript level, μ is the noise-free expression level, B is the background error following a Gaussian distribution (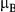, 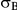) and η the multiplicative error following a Gaussian distribution (0, 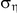). We simulated two different situations in terms of proportion π of expressed genes: a first one with equal proportions (π = 80%) on the X chromosome and on autosomes, and a second one with a lower proportion on the X than on autosomes (π = 60% vs 70%). To mimic real expression data, μ was sampled with probability π from the π% highest values of the empirical distribution of untransformed, background-corrected, expression levels of autosomal genes observed in GHS data, and was set to 0 with probability 1-π. Under the hypothesis of complete lack of dosage compensation, the value of μ for X-linked transcripts was multiplied by 0.5 to mimic the inactivation of one X copy (X∶AA = 0.5). We also simulated data under a model of partial compensation (X∶AA = 0.75) and full compensation (X∶AA = 1). Values for 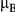 and 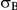 were taken from the empirical distribution of the negative controls provided by *Illumina* while 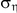 was estimated from the relation between the bead average expression and the bead standard error as in Lin et al. [16]. Simulated data were then transformed using the VST transformation and the X∶AA ratio was computed after filtering out either the lowest 50% of genes irrespective of the chromosome or the lowest 50% on the X chromosome and on autosomes separately. The simulation was repeated 10,000 times to generate confidence intervals.

All analyses were performed in R v. 2.10.1.

## Supporting Information

Figure S1 **Comparison of expression levels between the X chromosome and autosomes in human monocytes when selecting the most variable probe per gene (top, A: males, B: females) or the average of probe levels by gene (bottom, C: males, D: females).** The graph plots the X∶AA ratio of median expression of X-linked genes to autosomal genes according to the proportion of transcripts filtered out prior to analysis, using either a uniform threshold (red triangles) or individual thresholds on the X and on autosomes (green circles). Error bars show the 95% bootstrap confidence intervals. The horizontal dashed lines show the ratios expected if there was no dosage compensation (X∶AA = 0.5) or full compensation (X∶AA = 1).
(TIFF)

Figure S2 **Comparison of expression levels between the X chromosome and autosomes in mouse heart tissue.** (**A**) The graph plots the X∶AA ratio of median expression of X-linked genes to autosomal genes according to the proportion of transcripts filtered out prior to analysis, using either a uniform threshold (red triangles) or individual thresholds on the X and on autosomes (green circles). Error bars show the 95% bootstrap confidence intervals. The horizontal dashed lines show the ratios expected if there was no dosage compensation (X∶AA = 0.5) or full compensation (X∶AA = 1). (**B**) X∶AA ratios when the X is compared to individual autosomes and the same proportion of transcripts (50%) is filtered out on the X and on each autosome.
(TIFF)

Table S1 **List of the probes corresponding to imprinted genes in the GHS dataset.**
(XLS)
