# Supplementary figures and images for: The Choice of the Filtering Method in Microarrays Affects the Inference Regarding Dosage Compensation of the Active X-Chromosome
Source: PLoS One. 2011 Sep 1;6(9):e23956. doi: 10.1371/journal.pone.0023956 (PMC3164665; doi:10.1371/journal.pone.0023956)

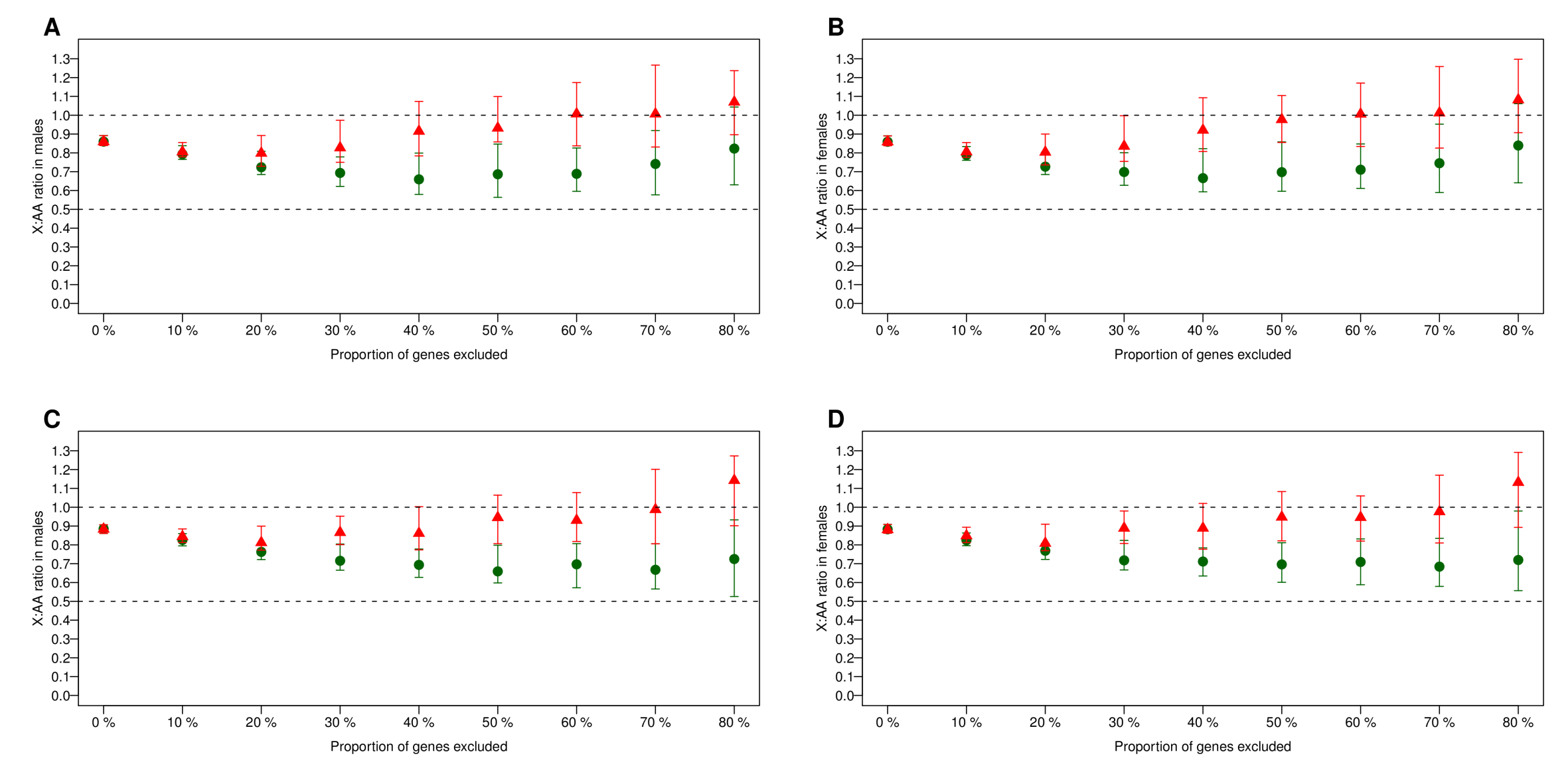

Supplement: Figure S1 — Comparison of expression levels between the X chromosome and autosomes in human monocytes when selecting the most variable probe per gene (top, A: males, B: females) or the average of probe levels by gene (bottom, C: males, D: females). The graph plots the X∶AA ratio of median expression of X-linked genes to autosomal genes according to the proportion of transcripts filtered out prior to analysis, using either a uniform threshold (red triangles) or individual thresholds on the X and on autosomes (green circles). Error bars show the 95% bootstrap confidence intervals. The horizontal dashed lines show the ratios expected if there was no dosage compensation (X∶AA = 0.5) or full compensation (X∶AA = 1). (TIFF) [file pone.0023956.s001.tiff]

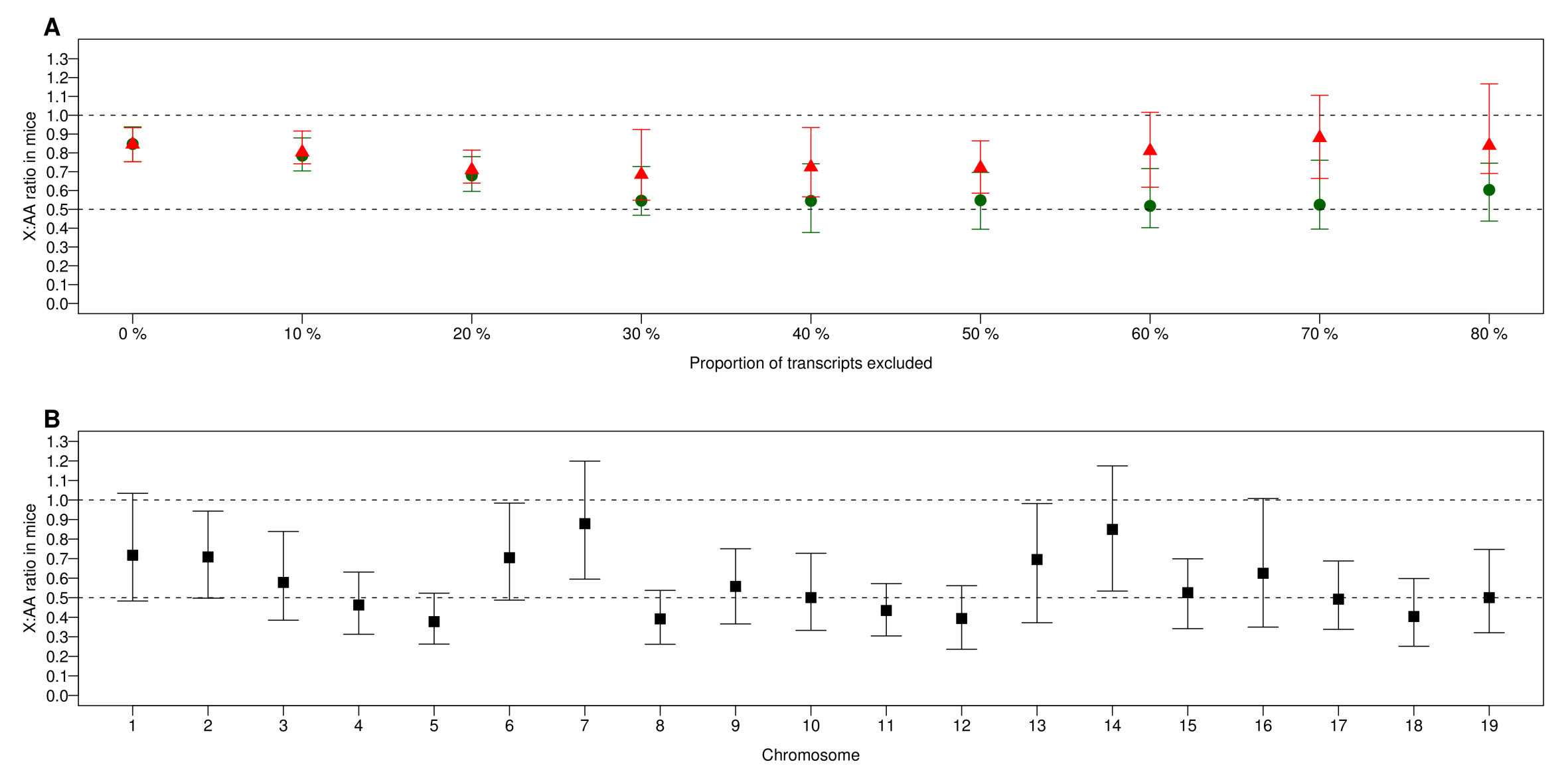

Supplement: Figure S2 — Comparison of expression levels between the X chromosome and autosomes in mouse heart tissue. (A) The graph plots the X∶AA ratio of median expression of X-linked genes to autosomal genes according to the proportion of transcripts filtered out prior to analysis, using either a uniform threshold (red triangles) or individual thresholds on the X and on autosomes (green circles). Error bars show the 95% bootstrap confidence intervals. The horizontal dashed lines show the ratios expected if there was no dosage compensation (X∶AA = 0.5) or full compensation (X∶AA = 1). (B) X∶AA ratios when the X is compared to individual autosomes and the same proportion of transcripts (50%) is filtered out on the X and on each autosome. (TIFF) [file pone.0023956.s002.tiff]
